# Adenoviral Vector-Based Vaccine Expressing Hemagglutinin Stem Region with Autophagy-Inducing Peptide Confers Cross-Protection Against Group 1 and 2 Influenza A Viruses

**DOI:** 10.3390/vaccines13010095

**Published:** 2025-01-20

**Authors:** Wen-Chien Wang, Ekramy E. Sayedahmed, Marwa Alhashimi, Ahmed Elkashif, Vivek Gairola, Muralimanohara S. T. Murala, Suryaprakash Sambhara, Suresh K. Mittal

**Affiliations:** 1 Department of Comparative Pathobiology, Purdue Institute of Inflammation, Immunology and Infectious Disease, College of Veterinary Medicine, Purdue University, 625 Harrison St., West Lafayette, IN 47907, USA; wang5382@purdue.edu (W.-C.W.); esayedah@purdue.edu (E.E.S.); alhashim@purdue.edu (M.A.); aelkashi@purdue.edu (A.E.); vgairola@purdue.edu (V.G.); mmurala@purdue.edu (M.S.T.M.); 2 Influenza Division, National Center for Immunization and Respiratory Diseases, Centers for Disease Control and Prevention, Atlanta, GA 30329, USA

**Keywords:** human adenoviral vector, bovine adenoviral vector, nonhuman adenoviral vector, adenoviral vector, hemagglutinin stem, extracellular domain of matrix protein 2, influenza vaccine, universal influenza vaccine, broadly protective influenza vaccine, autophagy, autophagy-inducing peptide, seasonal influenza vaccine, pandemic influenza vaccine

## Abstract

**Background/Objectives:** An effective universal influenza vaccine is urgently needed to overcome the limitations of current seasonal influenza vaccines, which are ineffective against mismatched strains and unable to protect against pandemic influenza. **Methods:** In this study, bovine and human adenoviral vector-based vaccine platforms were utilized to express various combinations of antigens. These included the H5N1 hemagglutinin (HA) stem region or HA2, the extracellular domain of matrix protein 2 of influenza A virus, HA signal peptide (SP), trimerization domain, excretory peptide, and the autophagy-inducing peptide C5 (AIP-C5). The goal was to identify the optimal combination for enhanced immune responses and cross-protection. Mice were immunized using a prime-boost strategy with heterologous adenoviral (Ad) vectors. **Results:** The heterologous Ad vectors induced robust HA stem-specific humoral and cellular immune responses in the immunized mice. Among the tested combinations, Ad vectors expressing SP + HA stem + AIP-C5 conferred significant protection against group 1 (H1N1 and H5N1) and group 2 (H3N2) influenza A viruses. This protection was demonstrated by lower lung viral titers and reduced morbidity and mortality. **Conclusions:** The findings support further investigation of heterologous Ad vaccine platforms expressing SP + HA stem + AIP-C5. This combination shows promise as a potential universal influenza vaccine, providing broader protection against influenza A viruses.

## 1. Introduction

Influenza viruses can result in severe respiratory infections and lead to approximately 290,000–650,000 deaths annually [1]. According to the Centers for Disease Control and Prevention (CDC) guidelines, using seasonal influenza vaccines is considered the best approach for influenza prevention [1]. The influenza virus hemagglutinin (HA) is the primary target immunogen of seasonal influenza vaccines since the neutralizing antibodies against the HA head domain can restrict the infection [2,3]. The current formulation of seasonal influenza vaccines is primarily based on HA quadrivalent formulation representing H1N1 and H3N2 subtypes of influenza A viruses and Victoria and Yamagata lineages of influenza B viruses [4,5]. However, the HA head domain undergoes continuous mutations, resulting in an antigen shift that leads to a mismatch between circulating and vaccine strains [6,7], thereby impacting vaccine efficacy [8,9]. Moreover, human infections with avian influenza viruses of H3, H5, H7, and H9 subtypes also emphasize the public health threat from zoonotic influenza viruses [10,11,12].

There is a pressing need for an effective universal influenza vaccine for both seasonal influenza and pandemic preparedness. One of the key strategies to develop broadly protective or universal vaccines is to stimulate robust immune responses against the conserved influenza antigens such as the HA stem region [13], extracellular domains of matrix protein 2 (M2e) [14], nucleoprotein (NP) [15,16], and matrix protein 1 (M1) [17]. Given that the HA head domain, due to repeated influenza exposure or vaccination, serves as an immunodominant antigen [18], redirecting immunity against subdominant conserved immunogen/s will be vital for developing a universal influenza vaccine.

Adenoviral (Ad) vector-based vaccine platforms are well established for their ability to elicit potent humoral and cell-mediated immune (CMI) responses. This efficacy is attributed to pathogen-associated molecular patterns (PAMPs) in Ad vaccines, which are able to activate both toll-like receptors (TLR)-dependent and independent pathways, enhancing innate immunity [19,20]. Notably, Ad vectors can be administered through the mucosal route, thereby inducing an enhanced antigen-specific mucosal immunity, which can be critical for protection against respiratory pathogens [21,22]. Currently, several Ad-vectored vaccines, including those targeting SARS-CoV-2, Ebola, and hepatitis C, are either licensed or are undergoing evaluation in clinical trials [23,24,25]. Our previous studies have demonstrated that autophagy-inducing peptide C5 (AIP-C5) from *Mycobacterium tuberculosis* can upregulate the autophagy-dependent antigen presentation in macrophage and dendritic cells, leading to enhanced cell-mediated immune (CMI) responses [26]. Additionally, AIP-C5 induced higher CMI responses in animals immunized with Ad vector vaccines for influenza [15] and SARS-CoV-2 [27].

To evaluate the potential of an Ad vaccine platform expressing the HA stem region for a broad influenza vaccine, we investigated various combinations of the HA2 or HA stem region, M2e, trimerization motif, HA signal peptide, excretory peptide, or AIP-C5 expressed in human or bovine Ad vectors for their immunogenicity and protective efficacy. Immunogenicity studies in mice revealed that Ad vectors expressing HA2 or HA stem region in various combinations generated variable levels of humoral and CMI responses against the HA2 region. Notably, prime-boost vaccination of mice with Ad vectors expressing the HA stem + HA signal peptide + AIP-C5 conferred broader protection against the homologous and heterosubtypic influenza viruses. These findings underscore the potential of the Ad vaccine platform expressing an innovative HA stem region design as a promising approach for developing a broadly protective influenza vaccine.

## 2. Materials and Methods

### 2.1. Cells and Viruses

The cell lines used in this study, BHH/F5 (bovine–human hybrid clone 3 cells [28] expressing bovine E1B region), BHH/F5-I-SceI (BHH/F5 cell line that expresses I-SceI endonuclease), and BHH2C (bovine–human hybrid clone 2C) [28], were developed in our lab. The 293 [human embryonic kidney cells expressing human Ad type 5 (HAd-5) E1 proteins] was obtained from ATCC: CRL-1573TM [29] and MDCK.2 (Madin–Darby canine kidney) was obtained from ATCC: CRL-2936TM. The 293Cre (293 cell line that expresses Cre recombinase) was kindly gifted by Professor Frank Graham, Department of Biology, McMaster University, Hamilton, Ontario, Canada [30]. They were cultured as monolayers in Dulbecco’s Modified Eagle’s Medium (DMEM) (Corning, Corning, NY, USA) containing 10% reconstituted fetal bovine serum (Hyclone, Logan, UT, USA) and gentamycin (50 mg/mL).

A/Vietnam/1203/2004(H5N1)-PR8/CDC-RG [VN/1203/04] was generated through reverse genetics (RG) in the A/Puerto Rico/8/1934(H1N1) [PR8] backbone having a deletion in the polybasic cleavage site of the HA gene segment and was utilized for the homologous virus challenge. A/Puerto Rico/8/1934(H1N1) and A/Hong Kong/1/68(H3N2) [HK68] were used for heterosubtypic challenges. Influenza viruses were propagated in embryonated hen eggs and titrated in MDCK cells.

### 2.2. Generation of Replication-Defective Ad Vectors

The HA2 (347 to 568 amino acid residues of HA) and M2e (2 to 24 amino acid residues of M2) derived from A/Vietnam/1203/2004(H5N1) were used as the model antigens for generating Ad vectors for Study #1. The HA2 derived from A/Vietnam/1203/2004(H5N1) and M2e from A/Vietnam/1203/2004(H5N1) and A/Anhui/1-YK_RG03/2013(H7N9) were used as the model antigens for generating Ad vectors for Study #2. The HA and M2e amino acid sequences of H5N1 or H7N9 were acquired from the NCBI GenBank (accession number: ADD97095.1, ADD97099.1, and AKU41057.1). For Study #1, gene cassettes (HA2-M2e, SP-HA2-M2e, or SP-HA2-M2e-Tri) containing the HA2 and M2e conjugated with or without HA signal peptide (SP) or GCN4 isoleucine zipper trimerization (Tri) motif [31] were used to generate HAd vectors. For Study #2, gene cassettes (EP-HAstem-C5, EP-HAstem-4M2e-C5, SP-HAstem-C5, or SP-HAstem-4M2e-C5) containing different combinations of the HA stem (the HA head region, 59 to 289 amino acid residues of HA, was replaced by the flexible short linker to generate the HA stem region) [32], four tandem M2e motif (H5-H7-H5-H7), immunoglobulin excretory signal peptides (EP) [33], and AIP-C5 (C5) for generating BAd and HAd vectors. For gene cassettes containing EP, the transmembrane cytoplasmic domain of the HA stem was removed. The H5N1 HA (FullHA) gene construct was also used as a positive control. All constructs were codon-optimized for rodents and synthesized by GenScript Biotech (Piscataway, NJ, USA).

The protocol for generating HAd vectors has been described previously [34]. Briefly, a Cre recombinase site-specific recombination technology was utilized to insert an appropriate gene cassette (HA2 + M2e, SP + HA2 + M2e, SP + HA2 + M2e + Tri, FullHA, EP-HAstem-C5, EP-HAstem-4M2e-C5, SP-HAstem-C5, or SP-HAstem-4M2e-C5) into the E1 region of HAd-ΔE1E3 (HAd-5 E1 and E3 deleted empty vector) to generate HAd vectors (HAd-HA2 + M2e, HAd-SP + HA2 + M2e, HAd-SP + HA2 + M2e + Tri, HAd-FullHA, HAd-EP-HAstem-C5, HAd-EP-HAstem-4M2e-C5, HAd-SP-HAstem-C5, or HAd-SP-HAstem-4M2e-C5). All the gene constructs were regulated by the human cytomegalovirus (CMV) promoter and the bovine growth hormone (BGH) polyadenylation signal. The recombinant virus exhibited cytopathic effects 7–10 days after transfection into 293Cre cells. The HAd vectors were propagated in 293 cells and titrated in BHH2C cells [28].

For the generation of bovine Ad (BAd) vectors (BAd-HA2 + M2e, BAd-SP + HA2 + M2e, BAd-SP + HA2 + M2e + Tri, BAd-FullHA, BAd-EP-HAstem-C5, BAd-EP-HAstem-4M2e-C5, BAd-SP-HAstem-C5, or BAd-SP-HAstem-4M2e-C5), we used the technology of I-SceI-mediated linearization [35] of infectious vector genome containing an appropriate gene cassette (SP + HA2 + M2e + Tri, FullHA, EP-HAstem-C5, EP-HAstem-4M2e-C5, SP-HAstem-C5, or SP-HAstem-4M2e-C5) in the E1 region of BAd-ΔE1E3 (BAd-3 empty vector with E1 and E3 deletions) after transfection of BHH/F5-I-SecI cells. The recombinant vectors exhibited cytopathic effects 7–10 days post-transfection. The BAd vectors were replicated in BHH/F5 cells and titrated in BHH/F5-I-SecI cells. A cesium chloride density gradient ultracentrifugation protocol was used for the purification of all HAd or BAd vectors [36].

### 2.3. Immunoblotting

Protein expression of all HAd and BAd vectors was assessed via immunoblotting as previously described [37]. To confirm the expression of HA2 or HA stem protein, an H5 HA-specific polyclonal antibody (Sino Biological, Wayne, PA, USA) was utilized.

### 2.4. Immunogenicity and Protection Studies in Mice

For immunogenicity and protection studies, six-to-eight-week-old BALB/c mice were purchased from Harlan Sprague Dawley, Indianapolis, IN, USA. The animals were left for one week for acclimatization before receiving the vaccination.

In Study #1, animals (10 mice/group) were vaccinated intranasally (i.n.) with 3 × 10^7^ PFU of HAd-M2e-HA2, HAd-SP-M2e-HA2, and HAd-SP-M2e-HA2-Tri or HAd-ΔE1E3. At three weeks post-immunization, 5 animals/group were euthanized to collect blood via retroorbital puncture and lung washes (prepared by homogenizing lungs in PBS as previously described [37]) for evaluating antigen-specific antibody responses. To determine cellular immune responses, the lungs, mediastinal lymph nodes (MLNs), and spleens were collected. The remaining mice (5 animals/group) were challenged via the i.n. route with 100 mouse infectious dose 50% (MID_50_) of A/Vietnam/1203/2004(H5N1)-PR8/CDC-RG. The mouse groups were euthanized three days post-challenge, and viral load was titrated from the lung samples.

In Study #2, mice (9 animals/group) were vaccinated i.n. with 10^7^ PFU of BAd vectors [BAd-FullHA-C5, BAd-EP-HAstem-C5, BAd-EP-HAstem-4M2e-C5, BAd-SP-HAstem-C5, BAd-SP-HAstem-4M2e-C5, or BAd-ΔE1E3]. Four weeks later, the mice received a booster dose of 10^8^ PFU of HAd vectors [HAd-FullHA-C5, HAd-EP-HAstem-C5, HAd-EP-HAstem-4M2e-C5, HAd-SP-HAstem-C5, HAd-SP-HAstem-4M2e-C5, or HAd-ΔE1E3] matching their priming antigen. Four animals from each group were anesthetized at two weeks post-boost, and the sample collection was the same as in Study #1. The remaining 5 mice/group were challenged via the i.n. route with 100 MID_50_ of A/Vietnam/1203/2004(H5N1)-PR8/CDC-RG. The mice were euthanized at three days post-challenge, and lung virus titers were measured.

In Study #3, mice (35 animals/group) were immunized i.n. with 10^8^ PFU of HAd vectors (HAd-SP-M2e-HA2-tri, HAd-SP-HAstem-C5, HAd-FullHA-C5, or HAd-ΔE1E3). The animal groups received a booster dose with 3×10^7^ PFU of BAd vectors (BAd-SP-M2e-HA2-tri, BAd-SP-HAstem-C5, BAd-FullHA-C5, or BAd-ΔE1E3) at three weeks post-first vaccine dose. The animals (5 mice/group) were euthanized three weeks post-boost, and the sample collection was the same as Study #1. The remaining animals (10 mice/group) were challenged via the i.n. route with 5 mouse lethal dose 50% (MLD_50)_ of A/Vietnam/1203/2004(H5N1)-PR8/CDC-RG, A/Puerto Rico/8/1934(H1N1), or A/HongKong/1/68(H3N2). Three days post-challenge, half of the animals (5 mice/group) were euthanized to assess lung virus titers, while the remaining animals (5 mice/group) were monitored for 14 days for morbidity (weight loss) and mortality (survival rate). Mice losing more than 20% of their initial body weight were euthanized and recorded as dead.

### 2.5. Enzyme-Linked Immunosorbent Assay (ELISA)

ELISA was conducted as described previously [37]. For coating of 96-well ELISA plates (Immulon 2HB flat bottom; Thermo Fisher Scientific, Waltham, MA, USA), recombinant HA protein of A/Vietnam/1194/2004 (Sino Biological, Houston, TX, USA) or the mixture of H5 and H7 M2e peptides were used.

### 2.6. ELISpot Assay

The ELISpot assay was conducted as described previously [38]. In brief, the splenocytes, MLNs, and lung MN cells were used for assessing interferon-gamma (IFN-γ) or interleukin-2 (IL-2) secretion via ELISpot assay. The cells were stimulated with the HA2 peptide array from the HA protein of A/Vietnam/1203/2004 (H5N1) (peptide 58–87, NR-18974, BEI Resources, Manassas, VA, USA). The number of spot-forming units (SFUs) was quantified by the AID EliSpot reader (Autoimmun Diagnostika, Straßberg, Germany).

### 2.7. Statistical Analyses

All statistical analyses were conducted by using GraphPad Prism software version 10.0. One-way ANOVA with Tukey post-test was used to analyze the statistical significance. The data are shown as mean ± standard deviation (SD), while * for *p* < 0.05, ** for *p* < 0.01, *** for *p* < 0.001, **** for *p* < 0.0001, and ns for no significance are denoted.

## 3. Results

### 3.1. Development, Immunogenicity, and Protection Efficacy of HAd Vector Expressing HA2 and M2e of H5N1 Influenza Virus

**Figure 1:**
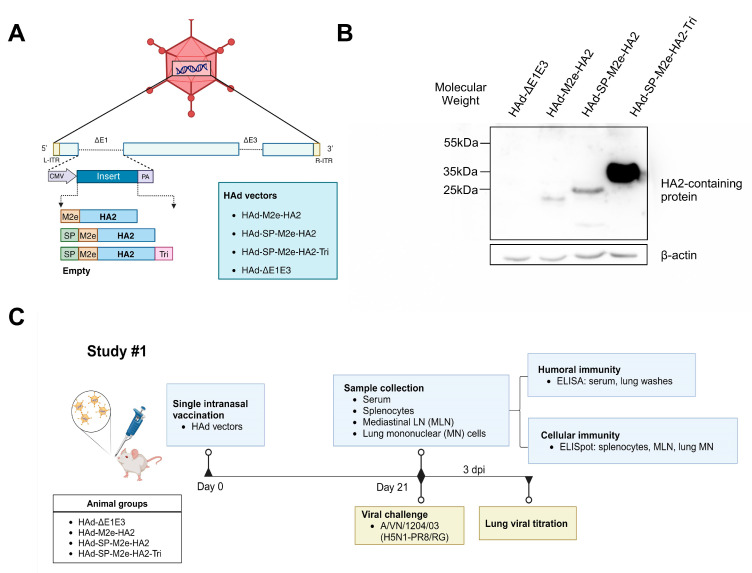
Diagrammatic representation of adenoviral (Ad) vector constructs and experimental plan of Study #1. (**A**) The expression cassettes containing the hemagglutinin domain 2 (HA2) gene of A/Vietnam/1203/2004(H5N1) conjugated with/without HA signal peptide (SP) and trimerization motif (Tri) were used for HAd5 (human Ad type 5) vectors. The resultant HAd vectors are listed. CMV, cytomegalovirus promoter; PA, bovine growth hormone (BGH) polyadenylation signal; ITR, inverted terminal repeats. (**B**) Expression of the HA2-containing protein in each Ad vector was confirmed by immunoblotting. Cell extract with empty vector infection was used as a negative control. (**C**) Eight-week-old BALB/c mice were vaccinated intranasally (i.n.) with a single dose of HAd vectors for the immunogenicity evaluation. Three weeks post-inoculation, mice were euthanized, and the blood, lungs, spleen, and mediastinal lymph node (MLN) were collected to evaluate the development of humoral and cell-mediated immune responses. To assess the protection efficacy, the mice were challenged with 100 mouse infectious dose 50% (MID_50_) of A/Vietnam/1203/2004(H5N1)-PR8/CDC-RG, and the lung viral titers were determined three days post-infection (dpi).

In Study #1, gene cassettes containing the HA2 and M2e regions from A/VN/1203/04 (H5N1) with or without SP or Tri were used to generate HAd-M2e-HA2, HAd-SP-M2e-HA2, and HAd-SP-M2e-HA2-Tri (Figure 1A). An empty vector, HAd-ΔE1E3, served as a negative control. The presence of the correct gene cassette in each vector was initially identified by restriction enzyme analysis of the genomic DNA followed by sequencing. To verify the protein expression, immunoblotting of HAd vector-infected cell extracts was performed using an anti-H5HA polyclonal antibody (Figure 1B). The presence of an approximately 25, 35, or 40 kDa band in HAd-M2e-HA2, HAd-SP-M2e-HA2, or HAd-SP-M2e-HA2-Tri-infected cell extracts, respectively, indicating successful expression of the target protein.

**Figure 2:**
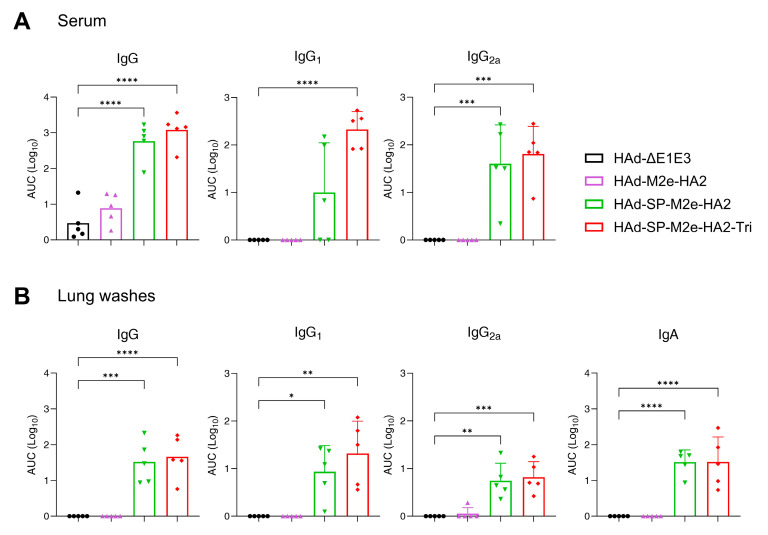
HA-specific antibody responses in the serum and lung washes of mice following adenoviral vector vaccine inoculation in Study #1. Eight-week-old BALB/c mice (5 animals/group) were vaccinated intranasally with 3 × 10^7^ PFU (plaque-forming units) of HA2-based HAd vectors as outlined in Figure 1C. Three weeks post-vaccination, the blood and lung washes were collected. The serum samples were used for determining HA-specific IgG, IgG_1_, and IgG_2a_ titers (**A**), while lung washes were examined for HA-specific IgG, IgG_1_, IgG_2a_, and IgA titers (**B**) using ELISA. The ELISA data are expressed in the area under curve (AUC) with a cut-off value determined by the average of blank wells. Each symbol represents an individual animal, and error bars indicate SD. Data were analyzed using one-way analysis of variance (ANOVA) with Dunnett’s post hoc test. Statistical significance compared to the empty vector group is denoted as follows: *, significant at *p* ≤ 0.05; **, significant at *p* ≤ 0.01; ***, significant at *p* ≤ 0.001; and ****, significant at *p* ≤ 0.0001.

The design of immunogenicity and protection studies are outlined (Figure 1C). Three weeks post-immunization with HAd vectors, samples from serum, lung wash, spleen, MLN, and lungs were collected to evaluate humoral and cellular immune responses. The serum and lung wash samples were used to assess the HA- or M2e-specific humoral immune responses by ELISA. In serum samples of HAd-SP-M2e-HA2- or HAd-SP-M2e-HA2-Tri-inoculated animals, there were inductions of significantly higher titers of HA-specific IgG, IgG_1,_ and IgG_2a_ antibodies compared to the empty vector (Figure 2A), while HAd-M2e-HA2 did not induce enough HA-specific antibodies. However, none of the HAd vectors induced significant M2e-specific IgG titers. In lung washes of HAd-SP-M2e-HA2- or HAd-SP-M2e-HA2-Tri-inoculated animals, there were significantly higher titers of HA-specific IgG, IgG_1_, IgG_2a,_ and IgA antibodies (Figure 2B), suggesting the induction of mucosal immunity. Again, none of the HAd vectors induced significant M2e-specific IgG titers in the lung washes.

**Figure 3:**
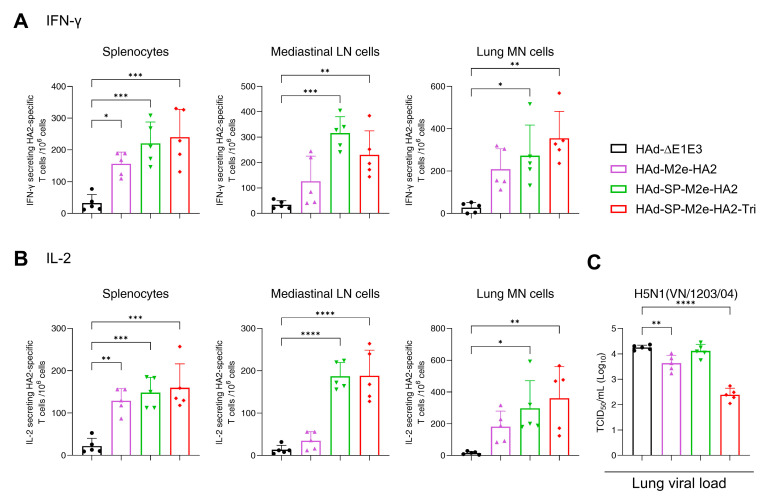
HA2-specific cellular immune responses and protective efficacy in mice vaccinated with adenoviral vector vaccines in Study #1. Eight-week-old BALB/c mice (5 animals/group) were inoculated intranasally (i.n.) with 3 × 10^7^ PFU (plaque-forming units) of HA2-based HAd vectors as shown in Figure 1C. At 3 weeks after vaccination, splenocytes, mediastinal lymph node (MLN) cells, and lung mononuclear (MN) cells were collected and stimulated with HA2 overlapping peptide array. The number of HA2-specific cytokine-expressing T cells was quantified using ELISpot assay. The number of HA2-specific T cells expressing IFN-γ (**A**) or IL-2 (**B**) in splenocytes, MLN cells, or lung MN cells is presented. To assess the protective efficacy, animals (5 animals/group) were challenged with 100 mouse infectious dose 50% (MID_50_) of A/Vietnam/1203/2004(H5N1)-PR8/CDC-RG influenza virus. Lung tissues were collected three days post-infection for viral load titration (**C**). Each symbol represents an individual animal, and error bars indicate SD. Data were analyzed using one-way analysis of variance (ANOVA) with Dunnett’s post hoc test. Statistical significance compared to the empty vector group is denoted as follows: *, significant at *p* ≤ 0.05; **, significant at *p* ≤ 0.01; ***, significant at *p* ≤ 0.001; and ****, significant at *p* ≤ 0.0001.

Splenocytes, MLN cells, and lung MN cells from immunized groups were analyzed using ELISpot assay to quantify IFN-γ- or IL-2-secreting T cells in response to the HA2 peptide array (Figure 3A,B). All three HAd vectors induced significantly higher amounts of INF-γ- or IL-2-secreting HA2-specific T cells in splenocytes compared to the empty vector. However, HAd-SP-M2e-HA2 or HAd-SP-M2e-HA2-Tri induced higher numbers of INF-γ- and IL-2-secreting HA2-specific T cells in MLN cells and lung MN cells, indicating the development of local CMI responses. Overall, both HAd-SP-M2e-HA2 and HAd-SP-M2e-HA2-Tri elicited robust HA2-specific systemic and local CMI responses.

To assess the protective efficacy of HA2-specific immune responses, vaccinated mice were challenged with VN/1203/04(H5N1)-PR8/CDC-RG, and lung viral titers were measured three days post-challenge. Mice vaccinated with HAd-SP-M2e-HA2-Tri or HAd-M2e-HA2 showed a significant reduction in lung viral titers (Figure 3C). However, no significant reductions in lung viral titers were observed in the HAd-SP-M2e-HA2-inoculated group. Based on immunogenicity and protection studies, HAd-SP-M2e-HA2-Tri was considered the best vaccine candidate in Study #1 and was used for further investigation in Study #3.

### 3.2. Development, Immunogenicity, and Protection Efficacy of BAd and HAd Vectors Expressing Headless HA (HA Stem) and Repetitive M2e Motifs of Influenza Virus

**Figure 4:**
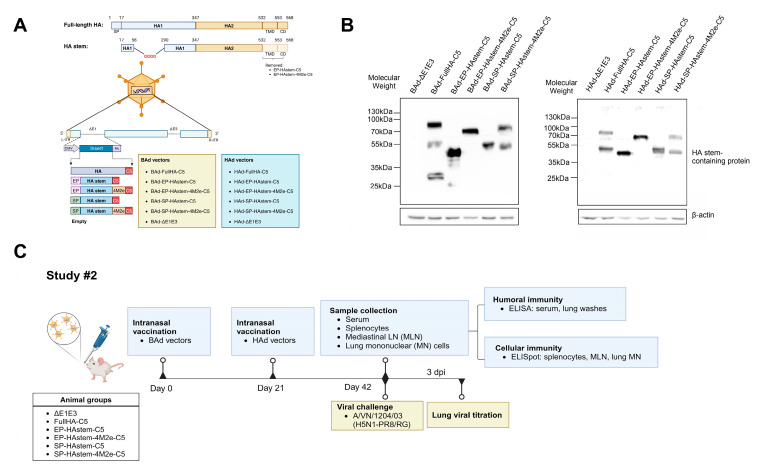
Diagrammatic representation of adenoviral (Ad) vector constructs and experimental plan of Study #2. (**A**) The expression cassettes containing the hemagglutinin stem region (HA stem) gene of A/Vietnam/1203/2004(H5N1) conjugated with immunoglobulin excretory signal peptides (EP) or HA signal peptide (SP) and with/without repetitive extracellular domains of matrix protein 2 (4M2e) were used for BAd3 (bovine Ad type 3) or HAd5 (human Ad type 5) vectors. An expressing cassette containing whole HA region (Full HA) was also generated. The resultant BAd and HAd vectors are listed. TND, transmembrane domain; CD, cytoplasmic domain; CMV, cytomegalovirus promoter; PA, bovine growth hormone (BGH) polyadenylation signal; ITR, inverted terminal repeats. (**B**) Expression of the HA stem-containing protein in each BAd and HAd vector was confirmed by immunoblotting. Cell extracts with empty vector infection were used as the negative control. (**C**) For the immunogenicity evaluation, 8-week-old BALB/c mice were vaccinated intranasally (i.n.) with BAd vectors and boosted with HAd vectors after 4 weeks. Two weeks after the second-dose inoculation, mice were euthanized, and the blood, lungs, spleen, and mediastinal lymph node (MLN) were collected to evaluate the development of humoral and cell-mediated immune responses. To assess the protection efficacy, the mice were challenged with 100 mouse infectious dose 50% (MID_50_) of A/Vietnam/1203/2004(H5N1)-PR8/CDC-RG, and the lung viral titers were determined three days post-infection (dpi).

In Study #2, to examine the effect of adding SP, EP, AIP-C5, and multiple domains of M2e to the HA stem, HAd (HAd-EP-HAstem-C5, HAd-EP-HAstem-4M2e-C5, HAd-SP-HAstem-C5, and HAd-SP-HAstem-4M2e-C5) and BAd (BAd-EP-HAstem-C5, BAd-EP-HAstem-4M2e-C5, BAd-SP-HAstem-C5, and BAd-EP-HAstem-4M2e-C5) vectors carrying EP-HAstem-C5, EP-HAstem-4M2e-C5, SP-HAstem-C5, or SP-HAstem-4M2e-C5 were generated, respectively. The FullHA-C5 gene cassette carrying HAd (HAd-FullHA-C5) and BAd (BAd-FullHA-C5) vectors were developed as positive controls. The gene construct organizations and their nomenclatures are shown (Figure 4A). The presence of the correct gene cassette in each vector was initially identified by restriction enzyme analysis of the genomic DNA followed by sequencing. To confirm the protein expression by HAd and BAd vectors, the immunoblotting of vector-infected cell extracts was performed using an anti-H5HA polyclonal antibody. The presence of an approximately 45, 65, 50, or 70 kDa band in HAd (HAd-EP-HAstem-C5, HAd-EP-HAstem-4M2e-C5, HAd-SP-HAstem-C5, and HAd-SP-HAstem-4M2e-C5) and BAd (BAd-EP-HAstem-C5, BAd-EP-HAstem-4M2e-C5, BAd-SP-HAstem-C5, and BAd-SP-HAstem-4M2e-C5) vectors (Figure 4B) suggested the expression of the target protein.

**Figure 5:**
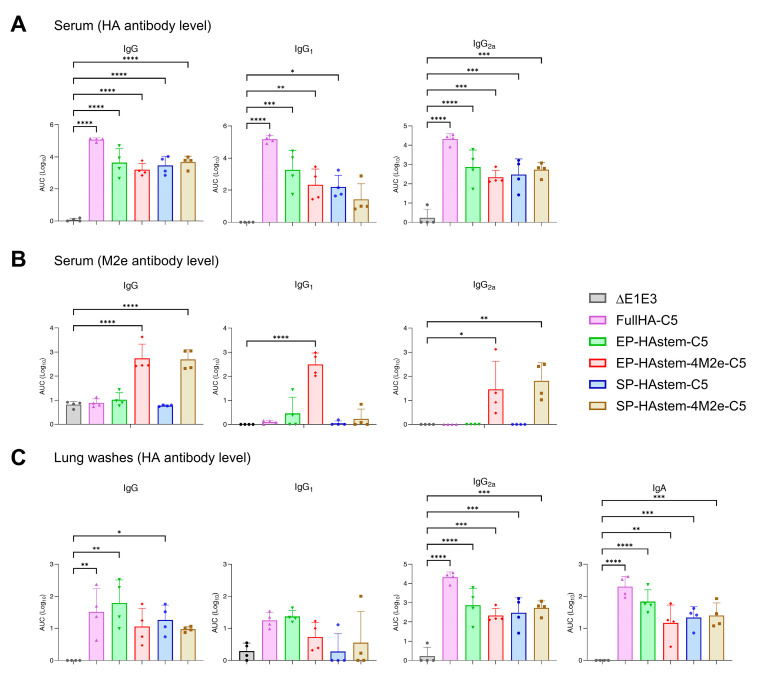
HA-specific antibody responses in the serum and lung washes of mice after adenoviral vector vaccine inoculation in Study #2. Eight-week-old BALB/c mice (4 animals/group) were vaccinated intranasally with 10^7^ PFU (plaque-forming units) of HA stem-based BAd vectors and boosted with 10^8^ PFU of HA stem-based HAd vectors as outlined in Figure 4C. The blood samples and lung washes were collected 2 weeks post-second dose. The serum samples were analyzed for HA-specific (A) or M2e-specific (B) IgG, IgG_1_, and IgG_2a_ titers, while lung washes were examined for HA-specific IgG, IgG_1_, IgG_2a_, and IgA titers (C) by ELISA. The ELISA data are expressed as the area under curve (AUC), with a cut-off value determined by the average of blank wells. Each symbol represents an individual animal, and error bars indicate SD. Data were analyzed using one-way analysis of variance (ANOVA) with Dunnett’s post hoc test. Statistical significance compared to the empty vector group is denoted as follows: *, significant at *p* ≤ 0.05; **, significant at *p* ≤ 0.01; ***, significant at *p* ≤ 0.001; and ****, significant at *p* ≤ 0.0001.

The immunogenicity and protection study outlines for Study #2 are depicted in Figure 4C. Mice were primed with BAd vectors and boosted with HAd vectors as described in the Material and Methods. The blood, lung wash, spleen, MLN, and lungs were collected three weeks post-booster immunization to evaluate humoral and cellular immune responses. The serum and lung wash samples were used to assess the HA- or M2e-specific humoral immune responses by ELISA. Vectors expressing FullHA-C5, EP-HAstem-C5, EP-HAstem-4M2e-C5, SP-HAstem-C5, or SP-HAstem-4M2e-C5 induced significantly higher titers of HA-specific IgG, IgG_1_, and IgG_2a_ antibodies than the empty vector (Figure 5A). Notably, vectors expressing multiple M2e domains (EP-HAstem-4M2e-C5 or SP-HAstem-4M2e-C5) induced significantly higher levels of M2e-specific IgG and IgG_2a_ antibodies (Figure 5B), indicating that the use of repetitive M2e motifs with prime-boost strategy enhanced the development of M2e-specific humoral immune responses. In addition, HA-specific IgG, IgG_2a,_ and IgA antibodies were detected in lung washes of immunized animal groups, suggesting the development of mucosal immunity (Figure 5C). Meanwhile, the higher induction of IgG_2a_ than IgG_1_ indicated a Th1-biased immune response.

**Figure 6:**
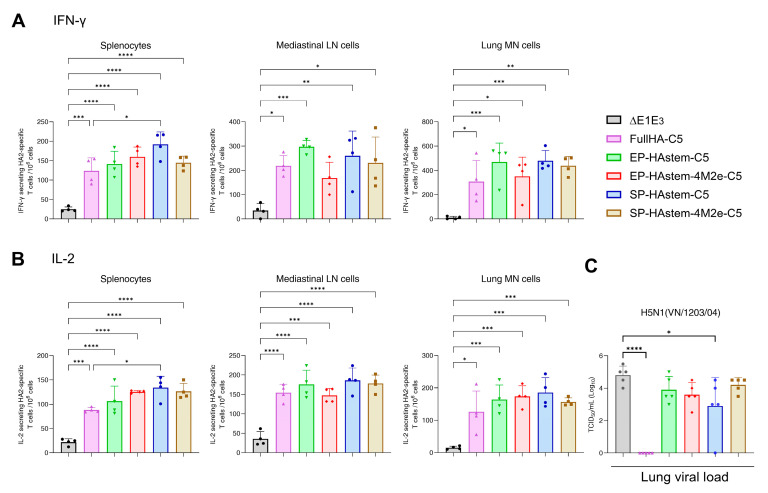
HA2-specific cellular immune responses and protective efficacy in mice vaccinated with adenoviral vector vaccines in Study #2. Eight-week-old BALB/c mice (4 animals/group) were vaccinated intranasally with 10^7^ PFU (plaque-forming units) of HA stem-based BAd vectors and received a booster with 10^8^ PFU of HA stem-based HAd vectors as shown in Figure 4C. Two weeks post-booster dose, splenocytes, mediastinal lymph node (MLN) cells, and lung mononuclear (MN) cells were collected and stimulated with an HA2 overlapping peptide array. The number of HA2-specific cytokine-expressing T cells was quantified using ELISpot assay. The number of HA2-specific T cells expressing IFN-γ (**A**) or IL-2 (**B**) in splenocytes, MLN cells, or lung MN cells are presented. To assess the protective efficacy, vaccinated animals (5 animals/group) were challenged with 100 mouse infectious dose 50% (MID_50_) of A/Vietnam/1203/2004(H5N1)-PR8/CDC-RG influenza virus. Lung tissues were collected three days post-infection for viral load titration (**C**). Each symbol represents an individual animal, and error bars indicate SD. Data were analyzed using one-way analysis of variance (ANOVA) with Dunnett’s post hoc test. Statistical significance compared to the empty vector group is denoted as follows: *, significant at *p* ≤ 0.05; **, significant at *p* ≤ 0.01; ***, significant at *p* ≤ 0.001; and ****, significant at *p* ≤ 0.0001.

To determine CMI responses, splenocytes, MLN cells, or lung MN cells from vaccinated groups were stimulated with an HA2 peptide array, and IFN-γ- or IL-2-secreting T cells were enumerated by ELISpot assay. All immunized groups showed significantly higher numbers of INF-γ and IL-2-secreting T cells in splenocytes, MLN cells, and lung MN cells compared to the empty vector (Figure 6A,B).

To evaluate the protection efficacy, immunized mouse groups were challenged with VN/1203/04(H5N1)-PR8/CDC-RG two weeks post-booster immunization, and the lung virus titers were measured. As expected, the vector expressing FullHA-C5 provided sterilizing immunity against the homologous virus (Figure 6C). In contrast, the mice vaccinated with the vector expressing SP-HAstem-C5 showed the most reduction in lung virus titers compared to other formulations expressing the HA stem (Figure 6C). Based on these findings, SP-HAstem-C5 was identified as the best candidate in Study #2; therefore, it was selected for further evaluation in Study #3.

### 3.3. Immunogenicity and Protection Efficacy of Mice Immunized with Prime-Boost Strategy Using HAd and BAd Vectors Expressing SP-M2e-HA2-Tri or SP-HAstem-C5

**Figure 7:**
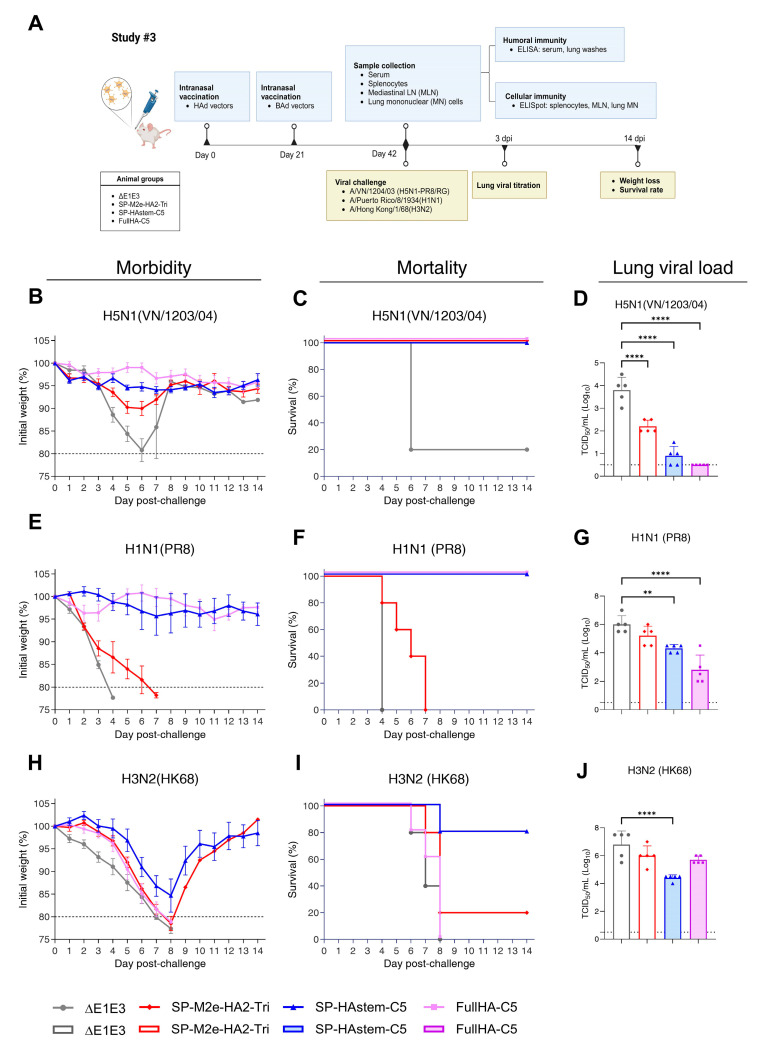
Experimental plan of Study #3 and morbidity, mortality, and lung viral load in the immunized mice challenged with homologous and heterosubtypic influenza viruses. (**A**) Vaccine candidates were selected based on the findings from Study #1 and Study #2. Eight-week-old BALB/c mice (5 animals/group) were immunized intranasally (i.n.) with HAd vectors, followed by a booster dose with BAd vectors at 3-week intervals. Immunized mice were challenged with 5 mouse lethal dose 50% (MLD_50_) of A/Vietnam/1203/2004(H5N1)-PR8/CDC-RG [VN/1203/04], A/Puerto Rico/8/1934(H1N1) [PR8], or A/HongKong/1/68(H3N2) [HK68] to evaluate the protection efficacy. The morbidity (weight loss) (**B**,**E**,**H**) and mortality (survival rates) (**C**,**F**,**I**) were monitored over 14 days post-challenge. The animals with weight loss exceeding 20% were euthanized and recorded as deceased. Data are shown as the mean with the error bars representing SD. The lungs were collected three days post-challenge for virus titration (**D**,**G**,**J**), expressed as Log_10_ TCID_50_ (50% tissue culture infectious dose), with a detection limit of 0.5 Log_10_ TCID_50_. Each symbol represents an individual animal, and error bars indicate SD. Data were analyzed using one-way analysis of variance (ANOVA) with Dunnett’s post hoc test. Statistical significance compared to the empty vector group is denoted as follows: **, significant at *p* ≤ 0.01; and ****, significant at *p* ≤ 0.0001.

The immunogenicity and protection results from Study #1 and Study #2 identified that the optimal antigen formulations were SP-M2e-HA2-Tri and SP-HAstem-C5, respectively. In addition, the prime-boost approach with HAd and BAd vectors indicated that it induced robust humoral and CMI responses. Therefore, BAd and HAd vectors expressing SP-M2e-HA2-Tri and SP-HAstem-C5 were selected for Study #3 to evaluate their protection efficacy against homologous and heterosubtypic influenza viruses. The BAd vector (BAd-SP-M2e-HA2-Tri) expressing SP-M2e-HA2-Tri was generated and characterized for the correct antigen expression (Supplementary Figure S1A). The empty vectors and vectors expressing FullHA-C5 were used as negative and positive controls, respectively. The outline of the immunogenicity and protection study in mice primed with HAd vectors and boosted with BAd vectors is illustrated in Figure 7A. The mouse groups immunized with vectors expressing SP-M2e-HA2-Tri, SP-HAstem-C5, or FullHA-C5 induced significantly higher titers of HA-specific IgG, IgG_1_, IgG_2a,_ and IgA antibodies in serum samples and lung washes compared to the empty vector (Supplementary Figure S1B,C); however, differences in antibody titers among vaccine groups were not statistically significant. Notably, the prime-boost approach did not induce significant M2e-specific IgG titers in the SP-M2e-HA2-Tri-vaccinated group, likely due to the presence of only a single M2e motif.

To evaluate CMI responses, splenocytes, MLN cells, or lung MN cells from vaccinated groups were stimulated with an HA2 peptide array for enumerating IFN-γ- or IL-2-secreting T cells by ELISpot assay. All immunized groups showed significantly higher numbers of INF-γ and IL-2-secreting T cells in splenocytes, MLN cells, and lung MN cells compared to the empty vector (Supplementary Figure S1D,E).

To assess the protection efficacy, immunized groups were challenged with VN/1203/04(H5N1)-PR8/CDC-RG, PR8 (H1N1), or HK68 (H3N2), and the morbidity, mortality, and lung virus titers were determined. Following the homologous VN/1203/04(H5N1)-PR8/CDC-RG challenge, no morbidity (weight loss) or mortality was observed in any vaccinated group, while the empty vector group displayed severe weight loss and 80% mortality (Figure 7B,C). All vaccinated groups also demonstrated significant reductions in lung virus titers compared to the empty vector group (Figure 7D). In the PR8 (H1N1) challenge, vectors expressing SP-HAstem-C5 or FullHA-C5 provided complete protection from morbidity and mortality. In contrast, significant morbidity and 100% mortality were observed in the SP-M2e-HA2-Tri or empty vector group (Figure 7E,F). Substantial reductions of lung virus titers were also observed in vectors expressing SP-HAstem-C5 or FullHA-C5 (Figure 7G). During the HK68 (H3N2) challenge, only the SP-HAstem-C5 vector conferred partial protection, resulting in approximately 15% weight loss on average and an 80% survival rate (Figure 7H,I). In contrast, there was significant morbidity and mortality in the SP-M2e-HA2-Tri, FullHA-C5, or empty vector group. In addition, there were substantial reductions of lung virus titers in the SP-HAstem-C5-vaccinated group compared to the empty vector group (Figure 7J). Overall, vectors expressing SP-HAstem-C5 provided broader protection across homologous, heterosubtypic group 1, and group 2 influenza A viruses, indicating its potential as a vaccine candidate for broad influenza protection.

## 4. Discussion

The current strategy for seasonal influenza vaccination predominantly relies on developing HA head-specific neutralizing antibodies, which are effective but have limitations [39]. However, the HA head domains of seasonal influenza viruses frequently mutate, altering their antigenic properties and thereby significantly reducing vaccine efficacy. Moreover, several avian influenza viruses have demonstrated their substantial risk as potential pandemic strains. Recent cases of avian A/H5N1 influenza virus (clade 2.3.4.4b) infecting cattle and spreading among cattle, humans, poultry, cats, and other wild mammals further underscore the potential danger of avian influenza viruses [40,41,42]. These implications highlight the critical global need to develop a broadly protective or universal influenza vaccine that offers better protection than current seasonal or pandemic influenza vaccines. Efforts have been made to target relatively conserved influenza antigens, such as the HA stem region, M2e domain, or NP, with varying degrees of success [15,43,44].

Unlike conventional influenza vaccine formulations, such as inactivated and subunit vaccines, which mainly induce humoral immune responses, Ad vector-based vaccines are considered a robust platform capable of stimulating both humoral and CMI responses [45]. We used the replication-defective BAd vector platform to circumvent the preexisting HAd vector immunity [46]. The BAd vector platform boosts innate immunity and triggers higher levels of CMI responses than the HAd vector system [37]. In addition, the BAd vector platform exhibits improved biodistribution to the lungs, enhancing its potential for delivering respiratory vaccines [47]. Furthermore, unlike many HAd or chimpanzee Ad vectors that use the coxsackievirus and adenovirus receptor (CAR) for cell entry, BAd3 exploits sialic acid-containing receptors, making it a better-suited vector for nasal delivery [48]. We used the prime-boost strategy with BAd and HAd vectors to boost antigen-specific immune responses.

The hepatic toxicity induced by Ad can be a concern of Ad vector-based vaccines. This issue is particularly prominent with HAd5 vectors administered intravenously at a high dose [49]. To address this, we utilized an intranasal route at a significantly low vector dose, which mitigates the risk of hepatic toxicity and effectively induces robust mucosal immunity. Another concern relates to vaccine-induced thrombotic thrombocytopenia (VITT), a rare condition primarily reported in individuals receiving AstraZeneca’s ChAdOx1 vaccine (a chimpanzee Ad-based SARS-CoV-2 vaccine). This condition may be due to the interaction between Ad hexon and platelet factor-4 (PF4), forming immune complexes that activate platelets and lead to thrombocytopenia [50]. However, the exact mechanism remains unclear, and there is ongoing debate about whether VITT is related to interactions with the SARS-CoV-2 spike protein [51]. Moreover, natural infections with HAds are not associated with VITT, and the use of Ad vector vaccines via the intranasal route, as in our study, may further mitigate the risk of this rare adverse event.

The HA stem domain of the influenza virus is essential for cell membrane fusion [52]. Unlike the more variable HA head region, the HA stem domain is relatively conserved due to lower immune pressure [6]. Monoclonal antibodies targeting the HA stem domain have demonstrated broad protective efficacy in mice, making it an ideal target for developing broadly protective influenza vaccines [13,53]. Various approaches have been employed to redirect the immune response toward the HA stem domain. For instance, sequential immunization with chimeric HAs (cHAs), consisting of exotic head domains with identical stem domains, can induce strong HA stem-specific immune responses [54]. The hyperglycosylation of the HA head region can also mask the antigenic sites from recognition by neutralizing antibodies, potentially enhancing the recognition of HA stem domain-specific epitopes [55]. In addition, headless HAs can be designed by removing the globular head domain to create modified HAs that include the HA1 and HA2 components of the stem domain [32]. Meanwhile, the HA stem domain has not naturally evolved to fold and trimerize independently without the presence of the head domain. Therefore, the modification has to be included to maintain the integrity of the HA stem region and the conformational epitopes [56,57]. In this study, we conducted two strategies to maintain trimer conformation in the HA2 or HA stem region. The trimerization motif was included with HA2 in Study #1 [58]. The replacement of the HA head domain with a flexible linker peptide was used to generate a headless HA stem in Study #2 [32].

The M2, derived from the gene segment 7 of the influenza virus, functions as a proton-selective ion channel and is vital in viral replication, morphogenesis, and assembly [59]. It comprises three regions: the N-terminal extracellular domain (M2e, residues 2–24), the transmembrane domain (residues 25–46), and the C-terminal intracellular domain (residues 47–97). Since the first nine amino acids of M2e share high similarity among influenza A viruses, it is considered a potential target for broadly protective influenza vaccine [60]. However, the main drawback of M2e is its low immunogenicity in natural infection and vaccination [14,61], likely due to steric hindrance by anti-HA antibodies, thereby restricting M2e’s accessibility to the immune system [62]. Here, we include a single M2e in Study #1, which did not induce any marked M2e-specific immune response. To address the drawback, we added repetitive M2e motifs to the C-terminal of the transgene constructs in Study #2, which strengthened the M2e-specific humoral immune responses. However, adding repetitive M2e did not result in significant protective immunity, and the construct without M2e showed better protection. Therefore, we excluded the constructs with repetitive M2e in Study #3.

SPs are short sequences located at the N-terminus of proteins that direct the proteins for cytoplasmic trafficking and are critical for protein expression [63]. A previous study demonstrated that the functional expression can be defective in vitro when the HA of an influenza virus lacks SP or has mutations in SP [64]. The exclusion of SP also negatively affects the stimulation of antibody and CD8^+^ T cell responses [65]. In Study #1, we observed a considerable decrease in protein expression and stimulation of immune responses without SP, consistent with the previous findings. In addition, the conjugation of SPs from different origins alters immunogenicity. It was demonstrated that the conjugation of SP from a secreted molecule can enhance immunogenicity in DNA vaccines compared to that from a transmembrane molecule [66]. The inclusion of the IgG SP also increases the cellular immunogenicity in a simian Ad-vectored vaccine [67]. Therefore, we hypothesized that replacing the original HA SP with immunoglobulin EP can induce a better immune response and evaluated the hypothesis in Study #2. However, there is no apparent difference between EP and SP in either protein expression or induction level of immune responses. In addition, we demonstrated that AIP-C5 can increase the expression of genes related to the autophagy pathway and can be utilized as a molecular adjuvant to facilitate autophagy and antigen presentation and induce more potent immune responses [26,68]. The expression of AIP-C5 can stimulate robust CMI responses in Ad vector-based vaccines against Mtb, SARS-CoV-2, or influenza virus [15,27,68]. Therefore, we included the AIP-C5 in the construct design to enhance the CMI responses.

In Study #3, immunization with Ad vectors containing HA2, HA stem, or whole HA provided complete protection against the homologous virus [A/Vietnam/1203/2004(H5N1)]. The SP-HAstem-C5 provided complete protection after the challenge with A/Puerto Rico/8/1934(H1N1), while severe weight loss and 100% mortality were observed in SP-M2e-HA2-Tri. Based on the HA phylogenetic characteristics, influenza A viruses can be categorized into two groups (group 1 includes H1, H2, H5, H6, H8, H9, H11, H12, H13, and H16, and group 2 includes H3, H4, H7, H10, H14, and H15). The HA stem-specific antibodies that provide cross-protection were usually observed within the same group [69,70]. It seems that H5N1 HA stem-specific immune responses stimulated by SP-HAstem-C5 can be cross-reactive toward the H1 influenza virus. Interestingly, the FullHA-C5 also provides complete protection against A/Puerto Rico/8/1934(H1N1). Since the amino acid identity of the HA1 region between the vaccine strain [A/Vietnam/1203/2004(H5N1)] and challenge strain [A/Puerto Rico/8/1934(H1N1)] is only 56.88% and HA2 region is 80.18%, it implies that the Ad vector platform can generate a different pattern of the immune responses targeting both HA1 and HA2 regions. Surprisingly, partial protection against A/HongKong/1/68(H3N2), which belongs to group 2 influenza viruses, was observed in SP-HAstem-C5-vaccinated animals, even though the amino acid identity of the HA2 region between the vaccine strain and challenge strain is only 50.45%. A trimeric H1 HA stem was able to induce cross-reactive antibodies against group 1 (H1 and H5) and 2 (H3) influenza virus strains in vitro [71]. Moreover, a trimeric HA stem with M2e epitope subunit vaccine also confers complete cross-protection against groups 1 and 2 influenza A viruses in mice [72]. Collectively, these studies and our results may indicate that the stem-directed immune responses can still confer cross-group protection, and conformational epitopes on the trimeric HA stem may be critical. However, noticeable weight loss and partial mortality are noticed in SP-HAstem-C5-immunized animals, suggesting that a further improvement of antigen design is needed for better protection.

## 5. Conclusions

An effective universal influenza vaccine is needed to address the drawbacks of current seasonal influenza vaccines and to prepare for the next influenza pandemic. Our work demonstrated that the expression of headless HA stem with the original SP and AIP-C5 utilizing prime-boost vaccination scheme using HAd and BAd vectors elicited robust HA2-specific humoral and CMI responses conferring broad protection against group 1 and 2 influenza A viruses. Further modifications in the antigen design with the assistance of protein structure prediction are required to build a better trimeric conformation with or without the repetitive M2e motifs. The durability of HA2-specific memory T and B cells and the longevity of protective immunity against different influenza viruses should be assessed to determine the practical use of such vaccine formulations.

## Data Availability

The data are included in the article and Supplementary Materials and are available upon request from the corresponding authors.

## Supplementary Materials

The following supporting information can be downloaded at https://www.mdpi.com/article/10.3390/vaccines13010095/s1, Figure S1: Adenoviral (Ad) vector constructs and immune responses in mice immunized with Ad vector-based vaccines in Study #3.
