# Supplementary material for: Adenoviral Vector-Based Vaccine Expressing Hemagglutinin Stem Region with Autophagy-Inducing Peptide Confers Cross-Protection Against Group 1 and 2 Influenza A Viruses
Source: Vaccines (Basel). 2025 Jan 20;13(1):95. doi: 10.3390/vaccines13010095 (PMC11769558; doi:10.3390/vaccines13010095)
Supplement: Supplementary file 1 [file vaccines-13-00095-s001.zip › vaccines-3377489-supplementary.pdf]

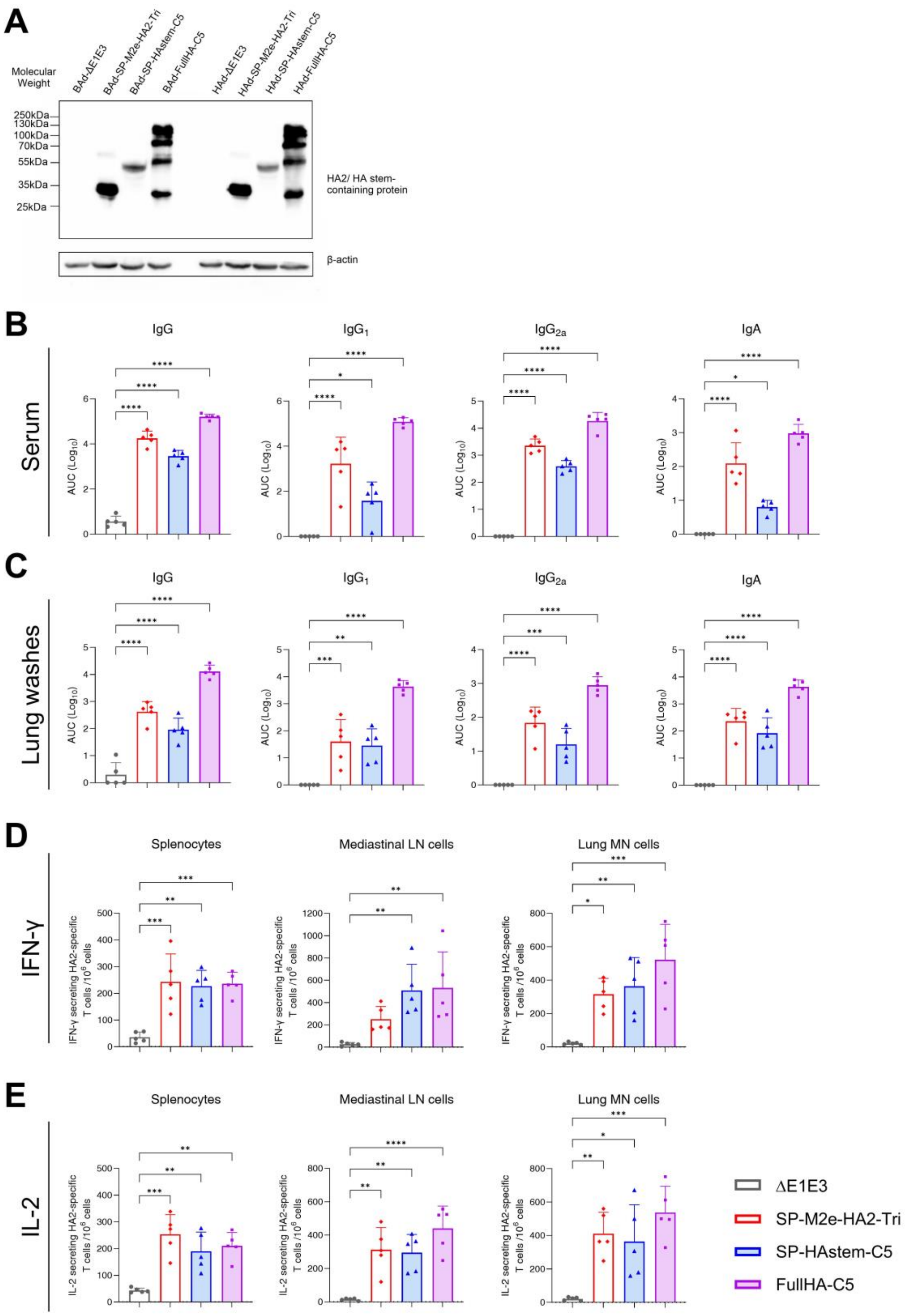

Supplementary Figure S1. Adenoviral (Ad) vector constructs and immune responses in mice immunized with Ad vector-based vaccines in Study #3. (**A)** The expression of the HA2 or HA stem by BAd and HAd vectors was validated by immunoblotting using vector-infected cell extracts. Mock-infected or empty vector-infected cell extracts served as negative controls. For immunogenicity, 8-week-old BALB/c mice (5 animals/group) were vaccinated intranasally (i.n.) with HAd vectors, followed by a booster inoculation with BAd vectors at the 3-week interval. Three weeks after the second dose, animals

were euthanized, and the blood, lungs, spleen, and mediastinal lymph node (MLN) were collected to assess humoral and cell-mediated immune responses. To evaluate the humoral immune responses, the serum samples **(B)** and lung washes **(C)** were analyzed for HA-specific IgG, IgG_1, IgG_{2a} and IgA titers using ELISA. The ELISA data were expressed as the area under curve (AUC), with cut-off value determined by the average of blank wells. Splenocytes, MLN cells and lung mononuclear (MN) cells were collected and stimulated with an HA2 overlapping peptide array to evaluate cellular immune responses. The number of HA2-specific cytokine-expressing T cells was quantified using ELISpot assay. The number of HA2-specific T cells expressing IFN-γ (**D**) or IL-2 **(E)** in splenocytes, MLN cells or lung MN cells are presented. Each symbol represents an individual animal and error bars indicate SD. Data were analyzed using one-way analysis of variance (ANOVA) with Dunnett's post-hoc test. Statistical significance compared to the empty vector group is denoted as follows: *, significant at $p \leq 0.05$; **, significant at $p \leq 0.01$; ***, significant at $p \leq 0.001$; and ****, significant at $p \leq 0.0001$.
